# Sibling Bullying: A Prospective Longitudinal Study of Associations with Positive and Negative Mental Health during Adolescence

**DOI:** 10.1007/s10964-021-01495-z

**Published:** 2021-09-30

**Authors:** Umar Toseeb, Dieter Wolke

**Affiliations:** 1 Department of Education, University of York, York, YO10 5DD UK; 2 Department of Psychology, University of Warwick, Coventry, CV4 7AL UK

**Keywords:** Sibling, Bullying, Longitudinal, Mental health, Wellbeing, Adolescence

## Abstract

Sibling bullying is associated with poor mental health outcomes, but the relevance of specific bullying roles remains unclear. Data from a population-based study (*n* = 17,157, 48% female) focusing on early (11 years), middle (14 years), and late (17 years) adolescence were analyzed. Associations between sibling bullying roles in early adolescence and positive and negative mental health outcomes in late adolescence were investigated. Generally, bullying, irrespective of role, was associated with poorer mental health outcomes in late adolescence. As the frequency of bullying victimization increased between early and middle adolescence so did the severity of mental health outcomes in late adolescence. The developmental trajectories of externalizing problems were influenced by bullying in early adolescence. Sibling bullying, irrespective of role, is associated with poor mental health outcomes.

## Introduction

Childhood and adolescence are particularly vulnerable periods for the deterioration of mental health. Problematic sibling relationships are a key modifiable risk factor that may play an important role in the development of mental health during adolescence (Bowes et al., 2014). It remains unclear whether sibling bullying in early adolescence is associated with *both* positive *and* negative mental health in the longer term and whether the developmental trajectories of mental health difficulties differ depending on the sibling bullying role (i.e., uninvolved, victim-only, bully-only, bully-victim). In the current article, data from a large population cohort study were used to investigate the longitudinal relationships between sibling bullying and, both, positive and negative mental health.

### Sibling Bullying

Sibling bullying is widespread in the general population. It is defined as “any unwanted aggressive behaviour(s) by a sibling that involves an observed or perceived power imbalance and is repeated multiple times or is highly likely to be repeated; bullying may inflict harm or distress on the targeted sibling, including physical, psychological, or social harm” (Wolke et al., 2015, p918). Half of all 11-year olds report being involved in sibling bullying in the recent past (i.e., picked or hurt at least once per week), either as a perpetrator, a victim, or both (Toseeb et al., 2018). This decreases to approximately a third by the time young people reach the age of 14 years (Toseeb et al., 2020b). Despite this, the severity of the problem is underestimated. Sibling bullying is perceived as less severe than peer bullying (Khan & Rogers, 2015) and is often normalized by family members (Straus et al., 2006) and society (Wiehe, 1997). Unlike peer bullying, sibling bullying is not yet recognized as a public health concern, which is problematic given the emerging evidence of its negative correlates (Dantchev et al., 2019).

The circumstances and personal characteristics that make young people more vulnerable to sibling bullying have been identified. A recent study investigated the role of child-level individual differences, parenting and parental characteristics, and structural family factors in sibling bullying involvement, either as a victim, perpetrator, or both (Toseeb et al., 2020a). Eleven-year-olds were asked to self-report whether they had hurt or picked on their siblings on purpose or whether their siblings had hurt or picked on them on purpose. It was found that child-level individual differences, such as sex, temperament, and emotional regulation abilities are the strongest predictors of sibling bullying. Structural family characteristics, such as birth order and number of siblings, were also found to be important but to a lesser extent. Parenting and parental characteristics, such as harsh parenting, also had some effect on sibling bullying involvement but fewer of these were related to sibling bullying, which echoes the results from previous work in a separate sample (Dantchev & Wolke, 2019).

The importance of distinguishing between bullying groups (i.e., victim-only, bully-only, bully-victim) is well established. Much of the rationale for this is rooted in the peer bullying literature. For example, a large cross-national study compared several outcomes for different peer bullying groups (Nansel et al., 2004). Those in the victim-only group had poorer emotional adjustment compared to those in the bully-only group whereas those in the bully-only group fared worse on alcohol use problems compared to those in the victim-only group. Similarly, bully-victims have more alcohol use problems than victims and more emotional adjustment problems compared to bullies. Bully-victims are more likely to be provocative, physically stronger, and assertive than those who are only victims (Schwartz et al., 1997). These characteristics are likely to influence sibling bullying too. For example, the age difference between siblings means that one sibling is likely to be physically stronger than the other. Alternatively, it may be that bully-victims and victims are less successful in responding to victimization whereas pure bullies are able to respond in a way that ceases any further victimization. The explanations for sibling bullying that have been taken from the peer bullying literature focus on the underlying motivation for bullying as dominance.

The motivation for engaging in sibling bullying may also be different than that for engaging in peer bullying. From an evolutionary perspective, siblings may compete for access to parental resources or to protect from the loss of resources when a new sibling arrives. The resource control theory suggests that differences amongst siblings foster competition for resources (Hawley, 1999). Therefore, whilst involvement in sibling bullying is an important consideration, the focus of the current article was to compare whether the sibling bullying role was important for both positive and negative mental health. In line with the resource control theory, bullies are likely to benefit from engaging in aggression as it is likely to result in continued access to parental resources such as time, attention, and affection and so may have better mental health outcomes compared to victims.

### Positive and Negative Mental Health

Theoretically, there is debate around whether positive and negative mental health are distinct constructs or whether they represent opposing ends of the same continuum. The term mental health is used in this article to broadly encompass both positive and negative mental health. The World Health Organization defines health as “a state of complete physical, mental, and social well-being and not merely the absence of disease or infirmity” (World Health Organisation, 1948, p2), thus emphasizing the importance of positive aspects of mental health rather than just the absence of negative mental health, which has been the focus of contemporary research. More specifically, *hedonic wellbeing* is concerned with the presence of positive affect (e.g., happiness and life satisfaction) rather than merely the absence of negative affect and *eudaimonic wellbeing* (e.g., self-esteem) includes striving for optimal functioning and self-actualization (Westerhof & Keyes, 2010). Negative mental health refers to mental health difficulties, defined as “a health condition involving changes in thinking, emotion or behaviour (or a combination of these) and is associated with distress and/or problems functioning in social, work or family activities” (American Psychiatric Association, 2013). Common childhood mental health difficulties include symptoms of psychiatric disorders such as depression, anxiety, and attention deficit hyperactivity disorder. The two-continua model of mental health posits that positive and negative mental health are related but distinct constructs (Westerhof & Keyes, 2010). Individuals without mental health difficulties do not always have high levels of wellbeing and, similarly, those with low levels of wellbeing do not necessarily experience mental health difficulties. Recent evidence from a population-based study of adolescents supports this assertion. Positive and negative mental health are only weakly related during adolescence and the correlates of the two are largely distinct highlighting the importance of considering them separately (Patalay & Fitzsimons, 2016).

### The Relationship between Sibling Bullying and Mental Health

There is extensive evidence linking sibling bullying to negative mental health. Cross-sectional studies in the United Kingdom (e.g., Toseeb et al., 2018) and elsewhere in the world, such as China (Liu et al., 2020), Portugal (Lopes et al., 2019), and the United States of America (Tucker et al., 2013), demonstrate that sibling bullying is associated with a wide range of mental health difficulties. There is also evidence for prospective longitudinal effects. Sibling bullying in childhood is associated with internalizing and externalizing problems in early adolescence (Toseeb et al., 2020b), psychotic disorder in late adolescence (Dantchev et al., 2018), and depression, self-harm, and suicidal ideation in late adolescence (Bowes et al., 2014) and in young adulthood (Dantchev et al., 2019), even after controlling for pre-existing mental health difficulties. Therefore, the current evidence suggests that there is a relationship between sibling bullying and mental health difficulties, both, cross-sectionally and longitudinally. The developmental course of mental health difficulties after sibling bullying remains unclear. That is, are the trajectories of change over time similar or different depending on the sibling bullying role (i.e., uninvolved, victim-only, bully-only, bully-victim).

The research evidence on the relationship between sibling bullying and positive mental health, in contrast, is scant. Two recent small-scale studies reported that higher levels of sibling bullying are associated with lower levels of life satisfaction (Plamondon et al., 2018) and self-esteem (Gan & Tang, 2020), but both relied on retrospective reports from adults about childhood sibling bullying. A larger population-based study in the United Kingdom reported cross-sectional associations between sibling bullying and life satisfaction, whereby higher levels of sibling bullying were associated with lower levels of life satisfaction in adolescence (Patalay & Fitzsimons, 2016). In the same cohort, a prospective longitudinal study reported associations between repeated sibling bullying at age 11 and 14 years and life satisfaction and self-esteem at age 14 years (Sharpe et al., 2021). Both studies are limited by their narrow focus on specific aspects of positive mental health (life satisfaction and self-esteem). Positive mental health is a multi-dimensional construct (Ruggeri et al., 2020) and so focusing on one aspect may limit understanding of the broader relationships with sibling bullying. Furthermore, there is so far no knowledge of the potential longer-term associations between sibling bullying and positive mental health, beyond the age of 14 years.

### The Effect of Repeated Sibling Bullying Victimization

The evidence regarding repeated sibling bullying victimization is starting to emerge. To the best of the authors’ knowledge only one longitudinal study has investigated the relationship between repeated sibling bullying victimization and mental health (Sharpe et al., 2021). It was found that adolescents who experienced repeated sibling bullying victimization between age 11 and 14 years had more mental health difficulties, lower life satisfaction, and lower self-esteem at age 14 years compared to those who did not. The study reported here builds on this work by investigating whether repeated sibling bullying victimization in early-to-mid adolescence is associated with positive and negative mental health in late adolescence (i.e., beyond age 14 years).

## Current Study

The relationship between sibling bullying in early adolescence and mental health in later adolescence remains unclear. Specifically, the prospective longitudinal associations between sibling bullying and positive (general wellbeing and self-esteem) and negative mental health (internalizing and externalizing problems, general psychological distress, and self-harm) need to be investigated separately. The current study addressed several aims. First, to investigate whether sibling bullying roles in early adolescence (i.e., uninvolved, victim-only, bully-only, bully-victim) are associated with positive and negative mental health in late adolescence. It was hypothesized that sibling bullying involvement at age 11 years will be associated with higher levels of mental health difficulties and lower levels of general wellbeing and self-esteem at age 17 years and these effects will differ depending on the sibling bullying role (Hypothesis 1). Second, to investigate whether there is a dose-response effect of sibling bullying victimization in early adolescence on positive and negative mental health in late adolescence. It was hypothesized that young people who are repeatedly bullied by siblings (i.e., at *both* age 11 *and* 14 years) will have higher levels of mental health difficulties and lower levels of wellbeing and self-esteem at age 17 years compared to those who are not bullied or only transiently bullied by siblings (i.e., *either* age 11 *or* 14 years) (Hypothesis 2). Finally, the study aimed to address the question of whether the developmental course of mental health difficulties (internalizing and externalizing problems) from early to late adolescence differs depending on the sibling bullying role in early adolescence. It was hypothesized that the trajectories of mental health difficulties between age 11 years and 17 years will differ depending on sibling bullying role at age 11 years (Hypothesis 3).

## Methods

### Sample

The Millennium Cohort Study (MCS) is an ongoing, multi-disciplinary study that follows the lives of approximately 19,000 children born in the United Kingdom between 2000 and 2002. See (Plewis, 2007a) and https://cls.ucl.ac.uk/cls-studies/millennium-cohort-study/ for full sampling details. These details are summarized here in brief. Families were identified as eligible for participation in the MCS using child benefit records, which were universal social security payments made to all families with children. Families were recruited to the study when the children were 9 months old and were subsequently followed up at age 3 years, 5 years, 7 years, 11 years, 14 years, and 17 years. Trained researchers administered surveys and conducted interviews in family homes at each wave of data collection. Parents answered questions about demographic characteristics (e.g., socioeconomic status indicators) and indicators of child health and well-being (e.g., physical activity, cognitive development, socioemotional well-being). During the latter stages of the study, young people themselves also completed self-report questionnaires and took part in assessments.

Participants for the current analysis (*N* = 17,157) were taken from the MCS, which is a population-based cohort study representative of the United Kingdom. The sex of the participants was approximately equally split (52% male). Participant ethnicity was also diverse (81% White, 10% South Asian, 4% Black, 3% Mixed, and 2% Other). For the analyses reported here, only the first child per family was included and those without any siblings at either age 11 or 14 years were excluded.

### Measures

#### Sibling bullying

At age 11 and 14 years, the young people were asked two questions about sibling bullying: “*how often do your brothers or sisters hurt you or pick on you on purpose?*” (victimization) and “*how often do you hurt or pick on your brothers or sisters on purpose?*” (perpetration). Responses were re-coded on to a six-point scale (0 = *never*, 1 = *less often*, 2 = *every few months*, 3 = *approximately once a month*, 4 = *approximately once a week*, 5 = *most days*). The correlation between a single-item scale, such as the one used here, and multi-item scales (e.g., Wolke & Samara, 2004) was calculated in an independent sample (Avon Longitudinal Study of Parents and Children (Boyd et al., 2013)), and it was shown to be high (victimization: *r* = 0.91, *n* = 6,909, *p* < 0.01; perpetration: *r* = 0.85, *n* = 6856, *p* < 0.01). There is also evidence to suggest that the prevalence of sibling aggression victimization and perpetration using single-item scales (e.g., Toseeb et al., 2018) is similar to that when using multi-item scales (e.g., Tippett & Wolke, 2015). Thus, there is good evidence for the validity of the single-item scales.

#### Self-report positive mental health

Adolescents self-reported two aspects of positive mental health when they were 17 years old.

##### General wellbeing

The seven-item Warwick-Edinburgh mental wellbeing scale (Tennant et al., 2007) was used to measure general wellbeing in the preceding two weeks. Sample questions were “I’ve been feeling optimistic about the future” and “I’ve been thinking clearly”. Responses to the seven questions were coded on a five-point scale (1 = *none of the time*, 2 = *rarely*, 3 = *some of the time*, 4 = *often*, 5 = *all of the time*). These were then summed and scaled in line with scoring guidelines so that higher scores indicated higher levels of wellbeing. The internal reliability for the scale was good (α = 0.83).

##### Self-esteem

The shortened five-item Rosenberg self-esteem scale (Rosenberg, 1965) was used to measures self-esteem. Sample questions were “on the whole, I am satisfied with myself” and “I am a person of value”. Responses to the five questions were recoded on to a four-point scale (0 = *strongly disagree*, 1 = *disagree*, 2 = *agree*, 3=*strongly agree*). These were then summed so that a higher score indicated higher levels of self-esteem. The internal reliability for the scale was excellent (α = 0.91).

#### Self-report negative mental health

Adolescents completed several well-validated measures of mental health difficulties when they were 17 years old.

##### Internalizing and externalizing problems

The self-report strengths and difficulties questionnaire (SDQ; Goodman, 1997) was completed by the young person with reference to the preceding six months. Responses were given on a three-point scale (0 = *not true*, 1 = *somewhat true*, 2 = *certainly true*). In line with the scoring guidelines (sdqinfo.org), four five-item subscales were created: emotional problems (e.g., “I get a lot of headaches, stomaches, and sickness”), peer problems (e.g., “I would rather be alone than with other people”), conduct problems (e.g., “I get very angry and often lose my temper”), and hyperactivity and inattention (e.g., “I am easily distracted, I find it difficult to concentrate”). In line with scoring guidelines and previous literature (e.g., Winsper et al., 2020), the emotional and peer problems subscales were combined to create an internalizing problems subscale and the conduct and hyperactivity subscales were combined to create an externalizing problems subscale. For all subscales, higher scores indicated more mental health difficulties. The internal reliability for both scales was acceptable (internalizing α = 0.74 and externalizing α = 0.75).

##### Psychological distress

The Kessler 6 scale (Kessler et al., 2003) was used to measure non-specific psychological distress. The scale consists of six questions relating to symptoms of depression and anxiety that the young person may have experienced in the preceding 30 days. Responses were re-coded on to a five-point scale (0 = *none of the time*, 1 = *a little of the time*, 2= *some of the time*, 3= *most of the time*, 4=*all of the time*). Sample items were “during the last 30 days, about how often did you feel so depressed that nothing could cheer you up?” and “during the last 30 days, about how often did you feel nervous?”. Responses were summed so that higher scores were indicative of higher levels of psychological distress. The internal reliability for the scale was good (α = 0.86).

##### Self-harm

Young people were asked whether they had hurt themselves on purpose in the preceding year. They were shown six types of self-harming behaviours and asked to respond on a binary scale (0 = *no*, 1 = *yes*). The behaviours were: “cut or stabbed yourself”, “burned yourself”, “bruised or pinched yourself”, “taken an overdose of tablets”, “pulled out your hair”, and “hurt yourself in some other way”. Responses were summed so that a higher score indicated higher levels of self-harm. The internal reliability for the scale was good (α = 0.81).

#### Parent-report negative mental health (internalizing and externalizing problems)

The primary caregivers (mostly the biological mother) completed a number of questionnaires about their child. The parent-report SDQ (Goodman, 1997) was completed by the primary caregiver about their child when the adolescent was 11, 14, and 17 years old. The parent-report version of the SDQ has identical questions to the self-report version described previously except the wording reflects its parent-report nature. Parents were asked to reflect on the preceding six months. As with the self-report version, emotional and peer problems items were combined to create an internalizing problems subscale. The conduct and hyperactivity items were combined to create an externalizing problems subscale. For all subscales, higher scores indicated more symptoms of mental health difficulties. The internal reliability for both scales was at least acceptable (internalizing problems α: 0.76 (11 years), 0.77 (14 years), 0.78 (17 years) and externalizing problems α: 0.81 (11 years), 0.81 (14 years), 0.80 (17 years)).

#### Covariates

A number of covariates were included in the statistical models. These are described in this section.

##### Pre-existing mental health difficulties

The parent-report strengths and difficulties questionnaire (SDQ: Goodman, 1997) was completed by the primary caregiver when the child was three years old. This has been described previously. The internal reliability of the scale was at least acceptable (internalizing α = 0.61 and externalizing α = 0.78).

##### Sex

At the first wave of data collection, primary caregivers reported their child’s biological sex (0 = *female*, 1 = *male*).

##### Poverty

Primary caregivers reported income from all sources (government benefits, employment etc.) when the young person was 11 years old, and this was used to calculate overall income. The OECD-modified scale was then used to standardize this overall household income (Hagenaars et al., 1994). Poverty was categorized as those families whose income was lower than 60% of the median income level (0 = *not in poverty*, 1 = *in poverty*).

### Statistical Analyses

STATA/MP version 17.0 (StataCorp, 2021) was used for data analysis. The analyses reported here were preregistered (https://osf.io/63q45). Some analyses, which were preregistered (research question 4 in the preregistration document), were removed from this study and will be included as part of a separate article. In addition to this, instead of using p-values to make inferences from the statistical models, 95% confidence intervals were used.

#### Missing data

There was some sample attrition over time. In line with the recommended use of the MCS dataset, the data were assumed to be missing at random (Plewis, 2007b). To maximize power, multiple imputation was used to deal with missing data. The proportions of missing data for each variable are shown in Table 1. The “mi impute” command with “chained” equations was used, which generated 50 imputed datasets. The command fills in missing values for multiple different variables with a set of possible values by using chained equations, a sequence of univariate imputation methods with fully conditional specification of prediction equations. Two imputation models were fitted, the first to test hypotheses 1 and 3 and the other to test hypothesis 2 (a single model was attempted but failed). To account for the application of disproportionate stratification and sample attrition all estimates were weighted to the population level. Weights were applied according to the MCS data handling guide (Agalioti-Sgompou & Johnson, 2020). Where possible, the “mibeta” command was used to calculate estimated standardized β coefficients.

**Table 1:** Missing data and imputed values

Variable	Complete	Imputed N	Imputed %	Total
Weighting variable	17,157	0	0%	17,157
Sex	17,157	0	0%	17,157
Poverty 11 years	11,313	5844	34%	17,157
Sibling bullying 11 years	10,818	6339	37%	17,157
Sibling bullying 14 years	9518	7639	45%	17,157
Positive mental health				
General wellbeing 17 years	8619	8538	50%	17,157
Self-esteem 17 years	8617	8540	50%	17,157
Negative mental health				
Internalizing problems 3 years (PR)	12,975	4182	24%	17,157
Internalizing problems 11 years (PR)	10,888	6269	37%	17,157
Internalizing problems 14 years (PR)	9557	7600	44%	17,157
Internalizing problems 17 years (PR)	7952	9205	54%	17,157
Externalizing problems 3 years (PR)	12,974	4183	24%	17,157
Externalizing problems 11 years (PR)	10,883	6274	37%	17,157
Externalizing problems 14 years (PR)	9556	7601	44%	171,57
Externalizing problems 17 years (PR)	7956	9201	54%	17,157
Internalizing problems 17 years	8395	8762	51%	17,157
Externalizing problems 1 7years	8395	8762	51%	17,157
Psychological distress 17 years	8624	8533	50%	17,157
Self-harm problems 17 years	8384	8773	51%	17,157

*PR* parent-report

In addition to the analyses reported in the main body of the article, a set of sensitivity analyses were carried out. For this additional set of analyses, all models were repeated with only participants with complete data on all four sibling bullying questions. This is reported in the supplementary materials (Tables S1–S3).

#### Testing hypothesis 1

Mutually exclusive sibling bullying groups were created based on established cut-offs (Wolke & Samara, 2004): victim-only: victimized at least once a week but not perpetrated; bully-only: perpetrated at least once a week but not victimized; bully-victim: both perpetrated and victimized at least once a week; uninvolved: does not meet the criteria for any of the other categories. To test whether sibling bullying at age 11 years is associated with self-report positive and negative mental health at age 17 years, six multiple regression models were fitted: internalizing problems (1), externalizing problems (2), psychological distress (3), self-harm (4), general wellbeing (5), and self-esteem (6). For each model, the predictor was entered as sibling bullying group (uninvolved, victim-only, bully-only, bully-victim) as a dummy variable with the uninvolved group as the reference category. The outcome was entered as one of the previously mentioned measures of positive and negative mental health. Sex, poverty, and pre-existing mental health difficulties were entered as covariates in all models. The models were then re-run each time changing the reference group.

#### Testing hypothesis 2

To determine whether there is a dose-response effect of sibling bullying victimization at age 11 and 14 years on self-report positive and negative mental health at age 17 years, six multiple regression models were fitted: internalizing problems (1), externalizing problems (2), psychological distress (3), self-harm (4), general wellbeing (5), and self-esteem (6). For each model, the predictor was entered as the victimization frequency (0 = *not bullied at least once per week at either age 11 or 14 years*, 1 = *bullied at least once per week at either age 11 or 14 years*, 2 = *bullied at least once per week at both age 11 and 14 years*). Initially, the reference category in all models was uninvolved. Therefore, the model estimates were for uninvolved vs transient and uninvolved vs repeated. Then, the reference category was changed to transient to allow for a comparison of the transient group to the repeated group. Sex, poverty, and pre-existing mental health difficulties were entered as covariates in all models.

#### Testing hypothesis 3

To determine whether the change in mental health difficulties between age 11 and 17 years was different depending on the type of sibling bullying involvement at age 11 years, two multilevel mixed-effects regression models were fitted. The outcome variable was entered as parent-report internalizing (1) or externalizing problems (2). The predictors in the fixed part of the model were the linear effect of age, sibling bullying group (uninvolved, victim-only, bully-only, bully-victim) - as a dummy variable with the uninvolved group as the reference category, and the interaction between sibling bullying group and linear effect of age. Anonymized participant number and the linear effect of age were included in the random part of the model. Sex, poverty, and pre-existing mental health difficulties were entered as covariates in all models. The models were then re-run each time changing the reference group to allow for comparisons of the three bullying groups to each other.

## Results

### Prevalence of Sibling Bullying

Descriptive statistics for the key variables are shown in Table 2. When the adolescents were 11 years old, 48% of the population sample were involved in at least one type of sibling bullying (victim-only 15%; bully-only 4%; bully-victim 29%). The remaining 52% were not involved in any type of sibling bullying. When they were 14 years old, 34% of the population sample were involved in at least one type of sibling bullying (victim-only 8%; bully-only 5%; bully-victim 21%). The remaining 66% were not involved in sibling bullying.

**Table 2:** Descriptive statistics for sibling bullying and mental health variables

	Range	Age 11 years	Age 14 years	Age 17 years
Sibling bullying perpetration frequency				
Never	–	2782 (26%)	3077 (32%)	–
Less often	–	2838 (26%)	2723 (29%)	–
Every few months	–	802 (7%)	522 (5%)	–
Approximately once a month	–	959 (9%)	747 (8%)	–
Approximately once a week	–	2172 (19%)	1502 (16%)	–
Most days	–	1361 (13%)	947 (10%)	–
Sibling bullying victimization frequency				
Never	–	2414 (22%)	3.274 (35%)	–
Less often	–	2255 (21%)	2384 (25%)	–
Every few months	–	647 (6%)	446 (4%)	–
Approximately once a month	–	802 (7%)	701 (7%)	–
Approximately once a week	–	2283 (21%)	1514 (16%)	–
Most days	–	2469 (23%)	1210 (13%)	–
Sibling bullying roles				
Uninvolved	–	5619 (52%)	6,334 (66%)	–
Victim-only	–	1698 (15%)	735 (8%)	–
Bully-only	–	470 (4%)	463 (5%)	–
Bully-victim	–	3031 (29%)	1986 (21%)	–
Positive mental health				
General wellbeing	7–35	–	–	22.47 (4.09)
Self-esteem	0–15	–	–	10.04 (3.21)
Negative mental health				
Internalizing problems (PR)	0–19	3.18 (3.12)	3.71 (3.4)	3.73 (3.43)
Externalizing problems (PR)	0–20	4.50 (3.60)	4.40 (3.60)	3.63 (3.31)
Internalizing problems	0–20	–	–	6.89 (4.88)
Externalizing problems	0–20	–	–	5.61 (3.30)
Psychological distress	0–24	–	–	7.17 (4.90)
Self-harm problems	0–6	–	–	0.42 (0.95)

Values are numbers before imputation. For categorical/ordinal variables values represent n (%). For continuous values represent mean (standard deviation). All variables were self-report except for those labelled as PR, which were parent-report

### Associations between Sibling Bullying and Positive and Negative Mental Health

To address the first aim, i.e., whether there is a relationship between sibling bullying roles (uninvolved, victim-only, bully-only, and bully-victim) at age 11 years and positive and negative mental health at age 17 years, a series of multiple regression models were fitted (Tables 3 and S4). ***Uninvolved vs Victim-only***. Those in the victim-only group at age 11 years had poorer outcomes across all mental health measures. They had more internalizing and externalizing problems, higher levels of psychological distress and self-harm, and lower levels of wellbeing and self-esteem at age 17 years compared to those in the uninvolved group. ***Uninvolved vs Bully-only***. Young people in the bully-only group at age 11 years had more externalizing problems and higher levels of psychological distress at age 17 compared to those in the uninvolved group. No other significant effects were observed for the bully-only group when compared to the uninvolved group. ***Uninvolved vs Bully-victim****.* For those in the bully-victim group at age 11 years, as with the victim-only group, they fared worse on all mental health measures at age 17 years compared to the uninvolved group. They had more internalizing and externalizing problems, higher levels of psychological distress and self-harming behaviors, and lower levels of wellbeing and self-esteem compared to those not involved in any sibling bullying at age 11 years. ***Victim-only vs Bully-only and Bully-Only vs Bully-victim****.* There were no differences in positive and negative mental health outcomes between the three sibling bullying groups. That is, those in the victim-only, bully-only, and bully-victim groups at age 11 years did not differ to each other in their mental health at age 17 years.

**Table 3:** The relationships between sibling bullying at age 11 years with self-report positive and negative mental health at age 17 years

	Internalizing problems	Externalizing problems	Psychological distress	Self-Harm problems	General wellbeing	Self-Esteem
Uninvolved [0] vs Victim-Only	**0.97 [0.62, 1.33] (0.07)**	**0.52 [0.27, 0.77]** **(0.06)**	**0.96 [0.60, 1.31] (0.07)**	**0.12 [0.05, 0.19]** **(0.04)**	**−0.83 [−1.11, −0.13] (−0.07)**	**−0.48 [−0.72, −0.23]** **(−0.05)**
Uninvolved [0] vs Bully-Only	0.51 [**−**0.07, 1.08] (0.02)	**0.99 [0.56, 1.42] (0.06)**	**0.80 [0.17, 1.43] (0.03)**	0.10 [**−**0.04, 0.24] (0.02)	**−**0.42 [**−**0.93, 0.10] (**−**0.02)	**−**0.10 [**−**0.54, 0.34] (**−**0.01)
Uninvolved [0] vs Bully-Victim	**0.69 [0.40, 0.98]** **(0.06)**	**0.73 [0.54, 0.92]** **(0.10)**	**0.74 [0.46, 1.02] (0.07)**	**0.11 [0.05, 0.16]** **(0.05)**	**−0.57 [−0.81, −0.34]** **(−0.06)**	**−0.33 [−0.54, −0.34]** **(−0.05)**
Victim-Only [0] vs Bully-Only	**−**0.47 [**−**1.13, 0.20] (**−**0.02)	0.47 [**−**0.01, 0.96] (0.03)	**−**0.16 [**−**0.85, 0.53] (**−**0.01)	**−**0.02 [**−**0.17, 0.14] (**−**0.00)	0.41 [**−**0.15, 0.97] (0.02)	0.37 [**−**0.08, 0.83] (0.02)
Victim-Only [0] vs Bully-Victim	**−**0.29 [**−**0.67, 0.10] (**−**0.03)	0.21 [**−**0.09, 0.50] (0.03)	**−**21 [**−**0.59, 0.16] (**−**0.02)	**−**0.01 [**−**0.09, 0.07] (**−**0.00)	0.25 [**−**0.07, 0.58] (0.03)	0.14 [**−**0.11, 0.40] (0.02)
Bully-Only [0] vs Bully-Victim	0.18 [**−**0.45, 0.81] (0.02)	**−**26 [**−**0.71, 0.18] (**−**0.04)	**−**0.06 [**−**0.73, 0.61] (**−**0.01)	0.01 [**−**0.13, 0.15] (0.00)	**−**0.16 [**−**0.72, 0.41] (**−**0.02)	**−**0.23 [**−**0.67, 0.21] (-0.03)

Bold font indicates confidence intervals that did not cross zero

Values are unstandardized beta [95% confidence interval] (standardized beta)

[0] is the reference category

All models include sex, poverty, and pre-existing mental health difficulties as covariates. For the internalizing problems pre-existing mental health difficulties were parent-report internalizing problems at age 3 years. For the externalizing problems model, pre-existing mental health difficulties were parent-report externalizing problems at age 3 years. For all other models, pre-existing mental health difficulties were entered as both parent-report internalizing and externalizing problems at age 3 years

Coefficients for covariates are shown in Table S4

These analyses were repeated using a multivariate regression model. This type of model assesses multiple dependent variables simultaneously, thus reducing the possibility of incorrectly rejecting a null hypothesis. This additional model confirmed the results of the separate regression models (see Table S5).

Therefore, the first hypothesis was partially supported. Being a victim-only and bully-victim in early adolescence was associated with poorer positive and negative mental health in late adolescence, when compared to those not involved in any sibling bullying. Being a bully-only in early adolescence, however, was only associated with increased externalizing problems and psychological distress in late adolescence, when compared to those not involved in any sibling bullying. There were, however, no differences in mental health outcomes between the three sibling bullying groups.

### The Effect of Transient and Repeated Sibling Bullying Victimization

To address the second aim, i.e., whether there is a dose-response effect of sibling bullying victimization at age 11 and 14 years on positive and negative mental health at age 17 years, several multiple regression models were fitted (Table 4 and Table S6). Those in the transient group (i.e., bullied at *either* age 11 *or* 14 years) had poorer outcomes on all measures of positive mental health and negative mental health compared to those in the uninvolved group. That is, adolescents who were victimized by siblings at *either* age 11 *or* 14 years (but not both) had more internalizing and externalizing problems, higher levels of psychological distress and self-harm, and lower levels of general wellbeing and self-esteem at age 17 years compared to adolescents who were not victimized by siblings at all. Similarly, those in the repeated group had poorer outcomes on all measures of positive and negative mental health at age 17 years compared to those in the uninvolved group. That is, adolescents who were victimized by siblings at *both* age 11 *and* 14 years had more internalizing and externalizing problems, higher levels of psychological distress, more self-harming behaviors, and lower levels of general wellbeing and self-esteem at age 17 years compared to those not victimized by siblings at either age 11 or 14 years. Additionally, outcomes for the transient group were compared with the outcomes for the repeated victimization group. Adolescents who were victimized by siblings repeatedly between age 11 and 14 years had poorer outcomes on all measures of positive and negative mental health at age 17 years compared to those who were victimized at *either* age 11 *or* 14 years. These results suggest that there may be a dose-response effect of sibling bullying victimization on later positive and negative mental health thus supporting the second hypothesis.

**Table 4:** The relationship between uninvolved, transient, and repeated sibling bullying victimization at age 11 and 14 years with self-report positive and negative mental health at age 17 years

	Internalizing problems	Externalizing problems	Psychological distress	Self-Harm problems	General wellbeing	Self-Esteem
Uninvolved [0] vs Transient	**0.77 [0.51, 1.03] (0.08)**	**0.64 [0.46, 0.82]** **(0.09)**	**0.70 [0.45, 0.97]** **(0.07)**	**0.07 [0.01, 0.12]** **(0.03)**	**−0.61 [−0.83, −0.39]** **(−0.07)**	**−0.28 [−0.46, −0.10]** **(−0.04)**
Uninvolved [0] vs Repeated	**1.53 [1.15, 1.90] (0.11)**	**1.27 [1.03, 1.51] (0.13)**	**1.90 [1.52, 2.25] (0.13)**	**0.24 [0.16, 0.32] (0.08)**	**−1.37 [−1.67, −1.07] (−0.11)**	**−0.82 [−1.07, −0.58]** **(−0.09)**
Transient [0] vs Repeated	**0.76 [0.40, 1.11]** **(0.05)**	**0.62 [0.37, 0.89]** **(0.06)**	**1.19 [0.81, 1.56]** **(0.08)**	**0.17 [0.09, 0.25]** **(0.06)**	**−0.75 [−0.1.04, −0.46]** **(−0.06)**	**−0.54 [0.10, −0.29]** **(−0.06)**

Values are unstandardized beta [95% confidence interval] (standardized beta). [0] is the reference category

Bold font indicates confidence intervals that did not cross zero

Values are unstandardized beta [95% confidence interval] (standardized beta)

[0] is the reference category

All models include sex, poverty, and pre-existing mental health difficulties as covariates. For internalizing problems pre-existing mental health difficulties refer to parent-report internalizing problems at 3 years. For externalizing problems pre-existing mental health difficulties refer to parent-report externalizing problems at 3 years. For all other models, pre-existing mental health difficulties refers to both parent-report internalizing and externalizing problems at 3 years

Coefficients for covariates are shown in Table S6

These analyses were repeated using a multivariate regression model. This type of model assesses multiple dependent variables simultaneously, thus reducing the possibility of incorrectly rejecting a null hypothesis. This additional model confirmed the results of the separate regression models (see Table S7).

### Sibling Bullying and the Trajectories of Mental Health Difficulties during Adolescence

To address the third aim, i.e., whether trajectories of mental health difficulties from age 11 to 17 years differ depending on sibling bullying roles at age 11 years, two multi-level mixed-effects models were fitted (one for parent-report internalizing problems and one for parent-report externalizing problems, Tables 5 and S8). Overall, boys had fewer internalizing problems and more externalizing problems than girls (main effect of sex). Those in poverty had more internalizing and externalizing problems compared to those not in poverty (main effect of poverty). Pre-existing mental health difficulties (i.e., internalizing or externalizing problems at age 3 years) were positively correlated with mean levels of internalizing and externalizing problems (main effect of pre-existing mental health difficulties).

**Table 5:** Sibling bullying roles and trajectories of parent-report mental health difficulties during adolescence

	Internalizing problems Unstandardized beta [95% CI]	Externalizing problems Unstandardized beta [95% CI]
Sibling bullying involvement group (Age 11)		
Uninvolved	0 [Reference]	0 [Reference]
Victim-only	**0.87 [0.33, 1.42]**	**1.30 [0.80, 1.80]**
Bully-only	0.65 [**−**0.37, 1.68]	**1.83 [0.81, 2.85]**
Bully-victim	**0.90 [0.42, 1.38]**	**1.88 [1.41, 2.35]**
Age	**0.12 [0.10, 0.14]**	**−0.09 [−0.11, −0.07]**
Sibling bullying involvement group × age interactions		
Uninvolved × Age	0 [Reference]	0 [Reference]
Victim-only × Age	**−**0.02 [**−**0.06, 0.02]	**−0.07 [−0.11, −0.04]**
Bully-only × Age	**−**0.03 [**−**0.10, 0.05]	**−0.08 [−0.15, −0.01]**
Bully-victim × Age	**−**0.03 [**−**0.07, **−**0.00]	**−0.10 [−0.13, −0.06]**
Sex		
Girls	0 [Reference]	0 [Reference]
Boys	**−0.25 [−0.37, −0.13]**	**0.82 [0.70, 0.95]**
Poverty		
No	0 [Reference]	0 [Reference]
Yes	**0.89 [0.73, 1.05]**	**1.04 [0.70, 0.95]**
Pre-existing mental health difficulties	**0.36 [0.33, 0.38]**	**0.36 [0.34, 0.38]**

Bold font indicates confidence intervals that did not cross zero

The models were repeated so that the mean and trajectories of all sibling bullying groups were compared to each other. These comparisons are shown in the Supplementary materials (see Table S8)

**Fig. 1:**
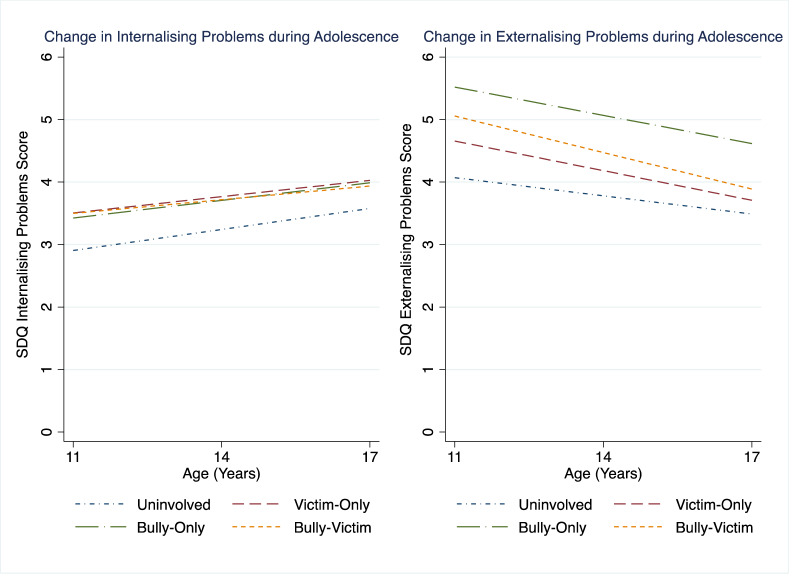
The developmental course of mental health difficulties after sibling bullying in early adolescence

For internalizing problems, being involved in sibling bullying as a victim-only and bully-victim at age 11 years (but not a bully-only), was associated with higher mean rates of difficulties compared to those not involved in any form of sibling bullying (main effect of sibling bullying involvement group). When the three sibling bullying groups (victim-only, bully-only, or bully-victim) were compared to each other, the mean rates of internalizing problems did not differ (see Table S8). There was an increase in internalizing problems between age 11 and 17 years (main effect of age) but this effect was not different depending on the sibling bullying group (sibling bullying involvement group X age interactions). That is, being involved in sibling bullying, irrespective of role, was associated with higher mean levels of internalizing problems between age 11 and 17 years compared to not being involved in any sibling. The trajectory of change in internalizing problems between age 11 to 17 years, however, was similar irrespective of whether young people were involved in sibling bullying or not (see Fig. 1).

The model for externalizing problems was slightly different. Adolescents who were involved in any type of sibling bullying at age 11 years (victim-only, bully-only, or bully-victim) had higher mean rates of externalizing problems overall (main effects of sibling bullying involvement group). When the three sibling bullying groups (victim-only, bully-only, or bully-victim) were compared to each other, the mean rates of externalizing problems did not differ, for the most part – there was a small difference between victim-only and bully-victim (see Table S8). Externalizing problems decreased over time (main effect of age) and this effect was different depending on the type of sibling bullying involvement at age 11 (sibling bullying involvement group X age interactions). The interaction terms for victim-only, bully-only, and bully-victim were significant, suggesting that the rate of decrease in externalizing problems between ages 11 and 17 years was faster for the three sibling bullying groups compared to the uninvolved group (see Fig. 1). The rate of change was not, however, different between the three sibling bullying groups.

## Discussion

Sibling bullying during childhood and adolescence is surprisingly common. It is associated with poor mental outcomes. The nature of the relationship between sibling bullying and *both* positive *and* negative mental health remains unclear. Data from a large population-based longitudinal cohort study were used to investigate whether different sibling bullying roles in early adolescence (uninvolved, victim-only, bully-only, bully-victim) are associated with diverse patterns of positive and negative mental health outcomes in late adolescence and whether the developmental trajectories of negative mental health were dependent on sibling bullying roles. The dose-response effect of sibling bullying on positive and negative mental health was also investigated. It was found that sibling bullying in early adolescence, irrespective of whether as a victim-only, bully-only, or bully-victim, is *generally* associated with higher levels of negative mental health and lower levels of positive mental health in late adolescence (with some exceptions). A dose-response effect of sibling bullying victimization was also observed; as the frequency of sibling bullying victimization increased between early and middle adolescence so did the severity of outcomes in late adolescence. Moreover, internalizing and externalizing problems between early and late adolescence were higher in those involved in sibling bullying, regardless of sibling bullying role. The rate of change in externalizing, but not internalizing, problems between early and late adolescence, differed depending on the sibling bullying role in early adolescence. These results are discussed with reference to relevant literature in the subsequent sections.

### Sibling Bullying and Positive and Negative Mental Health

The associations between sibling bullying and positive and negative mental health are, for the most part, similar. Sibling bullying involvement as a victim-only or bully-victim in early adolescence is associated with higher levels of negative mental health (i.e., mental health difficulties) and lower levels of positive mental health in late adolescence. These results are in line with expectations and support previous work on the prospective relationship between sibling bullying and mental health difficulties (e.g., Toseeb et al., 2020a). Similarly, sibling bullying involvement in early adolescence is associated with lower levels of positive mental health in late adolescence, which is in line with previous cross-sectional (e.g., Gan & Tang, 2020) and longitudinal research (e.g., Sharpe et al., 2021). The study extends previous work by focusing on two separate aspects of positive mental health (general wellbeing and self-esteem) in a single investigation. This is important given that positive mental health is a multi-dimensional construct (Ruggeri et al., 2020). The current study suggests that sibling bullying is similarly associated with both general wellbeing and self-esteem. Furthermore, to the best of the authors’ knowledge, this is the first study to focus on the prospective longitudinal associations between sibling bullying and general wellbeing adding to the literature demonstrating the possible long term detrimental effects of sibling bullying during early adolescence.

The results somewhat contrast results of previous work, which suggests that a much larger proportion of variance in negative mental health can be explained by common individual, family, and society level factors than positive mental health (Patalay & Fitzsimons, 2016). This previous study suggests that positive mental health may be much less malleable and less susceptible to external influences than negative mental health. There was no evidence to support this in the current study. The standardized effects sizes were similar for sibling bullying and positive (β = 0.05-0.07) and negative mental health (β = 0.03-0.10), which suggests that, at least in the longer term, if indeed causal, the magnitude of the influence of sibling bullying on positive and negative mental health is similar. Although significant, the effect sizes of sibling bullying were small (β < 0.1). However, as sibling bullying affects large numbers of adolescents any primary prevention that could shift the effect of sibling bullying to diminish would be highly significant in reducing mental health problems and increasing positive mental health in the population of young people.

Being a perpetrator but not a victim (i.e., a bully-only) of sibling bullying in early adolescence appears to have fewer effects on mental health outcomes in late adolescence. Those who bully their siblings but are not bullied by their siblings in early adolescence have more externalizing problems and psychological distress in late adolescence compared to those not involved in any sibling bullying, which is in line with expectations (e.g., Dantchev & Wolke, 2018). But on all other measures of positive and negative mental health, outcomes for pure bullies were comparable to those not involved in any sibling bullying. This effect (lack of) has been observed in some previous work (e.g., Toseeb et al., 2018) but not others (e.g., Liu et al., 2020).

This apparent lack of difference in mental health outcomes for pure bullies when compared to those not involved in any sibling bullying could be for several reasons. It may be that perpetrators of sibling bullying (those who are not also victims themselves) have higher levels of social cognition (Dantchev & Wolke, 2019). Evidence from peer bullying research suggests that bullies are less likely to have long term mental health problems (Copeland et al., 2013). Bullies have higher theory of mind skills, which allow them to understand others mental states and use this to their advantage (Sutton et al., 1999). More recent research suggests that bullies are neither superior nor deficient in the early stages of information processing but are less likely to have hostile attribution biases than victims (Guy et al., 2017). Indeed, social cognition skills, such as theory of mind, are impaired in those with common mental health disorders (Bora & Berk, 2016). Therefore, sibling bullies may indeed have social cognition skills that allow them to manipulate sibling relationships and protect their own mental health. This relationship between sibling bullying, social cognition skills, and mental health should be investigated to further understand the nature of these inter-relations. An alternative explanation is that the analysis was underpowered to detect an effect for the bully-only group. It was the smallest of the groups and the mental health outcomes were not different to any of the three other groups.

### The Effect of Sibling Bullying Role

The analyses reported here allowed for conclusions to be drawn about whether specific sibling bullying roles in early adolescence (i.e., victim-only, bully-only, bully-victim) were associated with specific positive and negative mental health outcomes in late adolescence. Whilst a different pattern of associations was observed for these groups when compared to the uninvolved group, when compared to each other, there were no differences in mental health outcomes. That is, mental health outcomes, both positive and negative, were not dependent upon the sibling bullying role in early adolescence. These results suggest that being involved in sibling bullying, irrespective of whether it is as a victim, perpetrator, or both, is associated with adverse mental health outcomes in late adolescence.

### Dose-Response Effect of Sibling Bullying Victimization

For the first time, it was possible to test whether sibling bullying victimization in early and middle adolescence is associated with both positive and negative mental health outcomes in late adolescence, in a dose-response manner. Being repeatedly victimized by siblings in both early and middle adolescence was associated with poorer positive and negative mental health in late adolescence when compared to those who were transiently victimized (i.e., bullied at *either* age 11 *or* 14 years) or not victimized at all. In addition to this, those who were transiently victimized had poorer outcomes compared to those who were not victimized at all. These results confirm previous work (Sharpe et al., 2021) and extend it by demonstrating that the dose-response relationship extends into late adolescence. This is important as it suggests that, if causality can be established, interventions aimed at reducing sibling bullying are likely to benefit both positive and negative mental health outcomes in late adolescence, even if not sibling bullying is not fully eliminated.

### Sibling Bullying and Trajectories of Mental Health Difficulties

This is the first study to investigate how mental health difficulties develop after sibling bullying involvement and how this differs depending on the sibling bullying role (i.e., uninvolved, victim-only, bully-only, bully-victim). It was found that parent-report internalizing and externalizing problems follow different patterns from early to late adolescence.

The mean levels of internalizing problems across early, middle, and late adolescence were higher in those who were victim-only or bully-victims compared to those not involved in any sibling bullying but were not different to each other or the bully-only group. Contrary to expectations, however, patterns of increase in internalizing problems from early to late adolescence did not differ between sibling bullying roles (i.e., uninvolved, victim-only, bully-only, bully-victim). That is, the growth in internalizing problems are similar irrespective of the sibling bullying role and the between-group differences are stable. Internalizing problems increase uniformly between early and late adolescence irrespective of sibling bullying involvement in early adolescence. This is not in line with expectations based on the developmental cascades framework (Masten & Cicchetti, 2010). The framework predicts that adverse experiences, such as sibling bullying, will have cumulative effects that snowball and cascade into other areas of functioning (like a downward spiral). For example, it might have been expected that sibling bullying leads to impaired development of social skills having an adverse effect on friendships, which are known to be protective against mental health difficulties (van Harmelen et al., 2016). The results for internalizing problems do not support this expectation. There is no evidence to suggest negative developmental cascades for sibling bullying on internalizing problems during adolescence. Rather, the results appear consistent that normative changes in internalizing scores during adolescence are related to multiple dimensions of maturation in the hypothalamic-pituitary-gonadal axis but the levels are associated with sibling bullying experience (Angold & Costello, 2006).

For externalizing problems, however, the decrease from early to late adolescence was faster for all three bullying groups compared to the uninvolved group but the three bullying groups did not differ to each other in the rate of decrease. The magnitude of the between-group differences appears to be larger in early adolescence than in late adolescence, although this was not tested directly (see Fig. 1). This suggests that, if indeed the relationship between sibling bullying and externalizing problems is causal, then the negative effects of sibling bullying become less pronounced over time. Again, this does not support expectations based on the developmental cascades framework (Masten & Cicchetti, 2010), whereby sibling bullying would lead to a negative cascade of adverse outcomes. One may speculate that sibling bullying victimization builds some resilience allowing adolescents to develop strategies to manage externalizing problems (Rutter, 2013) and so as they progress through adolescence, even though they consistently have more externalizing problems, these problems decrease at a faster rate compared to those not involved in any sibling bullying.

### Strengths and Limitations

A key strength of the analyses reported here is the use of data from a large representative sample. This allows for inferences to be made about what the results mean for the general population of the United Kingdom. Furthermore, well-validated and widely used measures of mental health difficulties and wellbeing were used allowing for comparisons to be made to other published research. Mental health was reported by both parents and young people themselves with similar results controlling for shared variance of the same data source. Whilst these are considerable strengths of the study, several limitations should also be borne in mind. The parent-report measure of mental health difficulties may be limited. In terms of internalizing problems, parents may be less aware and less able to accurately report how their child is feeling. Indeed, at age 17 years, the factor structure of the parent-report SDQ shows less than satisfactory fit in this sample (Murray et al., 2021). Furthermore, the peer problems subscale of the SDQ was included as part of internalizing problems, even though the items may be indicative of non-specific symptoms that are generalizable to externalizing problems. Although significant, the reported effect sizes are very small, meaning that, if causal, the effect of sibling bullying on mental health is small. Causal inferences cannot be made from the analyses reported. Despite longitudinal data being used, the cross-lagged effects of sibling bullying and mental health were not investigated. Therefore, the possibility that pre-existing mental health difficulties make adolescents more susceptible to sibling bullying cannot be ruled out.

## Conclusion

Sibling bullying, irrespective of whether as a victim, perpetrator, or both in early adolescence is associated with poorer positive and negative mental health in late adolescence. There is a dose-response effect of sibling bullying victimization on subsequent positive and negative mental health. Meaning that as the persistence of sibling bullying victimization increases so does the strength of the associations with various mental health outcomes. Adolescents who are involved in sibling bullying, irrespective of whether as a victim, perpetrator, or both follow a different trajectory of externalizing problems compared to those not involved in sibling bullying. If replicated using causal methods, these results suggest that sibling bullying in early adolescence has a long-term effect on both positive and negative mental health in late adolescence. Prevention and clinical interventions aimed at reducing mental health difficulties and promoting positive mental health during late adolescence are likely to benefit from reducing sibling bullying in early adolescence.

## Supplementary Information

Supplementary Materials
