# Supplementary material for: Sibling Bullying: A Prospective Longitudinal Study of Associations with Positive and Negative Mental Health during Adolescence
Source: J Youth Adolesc. 2021 Sep 30;51(5):940–55. doi: 10.1007/s10964-021-01495-z (PMC8993709; doi:10.1007/s10964-021-01495-z)
Supplement: Supplementary file 1 — Supplementary Materials [file 10964_2021_1495_MOESM1_ESM.docx]

**Supplementary Materials**

**Table S1**

*Sensitivity Analysis for the Relationships Between Sibling Bullying at age 11 Years with Self-Report Positive and Negative Mental Health at age 17 years*

|  |  | **Victim-Only** | | **Bully-Only** | | **Bully-Victim** | |
| --- | --- | --- | --- | --- | --- | --- | --- |
| **Outcome** | **Uninvolved** | **Unstandardized β [95% CI]** | **Standardized β** | **Unstandardized β [95% CI]** | **Standardized β** | **Unstandardized β [95% CI]** | **Standardized β** |
| Internalizing problems | Reference | .**94 [.63, 1.25]** | .07 | .40 [-.16, .97] | .02 | **.66 [.41, .92]** | .06 |
| Externalizing problems | Reference | **.57 [.35, .79]** | .06 | **.97 [.58, 1.37]** | .06 | **.71 [.54, .90]** | .10 |
| Psychological distress | Reference | **.93 [.62, 1.25]** | .07 | **.68 [.11, 1.25]** | .03 | **.81 [.55, 1.06]** | .08 |
| Self-harm problems | Reference | **.12 [.05, .19]** | .04 | .10 [-.04, .24] | .02 | **.11 [.05, .16]** | .05 |
| General wellbeing | Reference | **-.79 [-1.05, -.53]** | -.07 | **-.63 [-1.11, -.15]** | -.03 | **-.58 [-.80, -.36]** | -.07 |
| Self-esteem | Reference | **-.56 [-.77, -.35]** | -.06 | -.15 [-.54, .23] | -.01 | **-.36 [-.53, -.19]** | -.05 |

**Note.** Bold font indicates confidence intervals that did not cross zero. All models include sex, poverty, and pre-existing mental health difficulties as covariates. For the internalizing problem pre-existing mental health difficulties were parent-report internalizing problems at age 3 years. For the externalizing problems model pre-existing mental health difficulties was parent-report externalizing problems at age 3 years. For all other models, pre-existing mental health difficulties were entered as both parent-report internalizing and externalizing problems at age 3 years.

**Table S2**

*Sensitivity Analysis for the Relationship Between Transient and Repeated Sibling Bullying Victimization at age 11 and 14 years with Self-Report Positive and Negative Mental Health at age 17 years*

|  | **Uninvolved vs Transient** | | **Uninvolved vs Repeated** | | **Transient vs Repeated** | |
| --- | --- | --- | --- | --- | --- | --- |
| **Outcome** | **Unstandardized β [95% CI]** | **Standardized β** | **Unstandardized β [95% CI]** | **Standardized β** | **Unstandardized β [95% CI]** | **Standardized β** |
| Internalizing problems | **.64 [.40, 88]** | .06 | **1.46 [1.16, 1.76]** | .11 | **.82 [.51, 1.14]** | .06 |
| Externalizing problems | **.63 [.46, .80]** | .09 | **1.18 [.97, 1.39]** | .14 | **.56 [.34, .77]** | .06 |
| Psychological distress | **.58 [.34, .82]** | .06 | **1.91 [1.60, 2.22]** | .15 | **1.34 [1.02, 1.65]** | .10 |
| Self-harm | **.06 [.01, .11]** | .03 | **.24 [.18, .30]** | .10 | **.18 [.12, .25]** | .07 |
| General wellbeing | **-.51 [-.71, -.31]** | -.07 | **-1.32 [-1.57, -1.06]** | -.12 | **-.81 [-1.07, -.54]** | **-**.08 |
| Self-esteem | **-.23 [-.39, -.07]** | -.03 | **-.80 [-1.01, -.59]** | -.09 | **-.57 [-.79, -.36]** | -.07 |

**Note.** Bold font indicates confidence intervals that did not cross zero. All models include sex, poverty, and pre-existing mental health difficulties as covariates. Values for all co-variates are shown in the supplementary. For internalizing problems pre-existing mental health difficulties refers to parent-report internalizing problems at 3 years. For externalizing problems pre-existing mental health difficulties refers to parent-report externalizing problems at 3 years. For all other models, pre-existing mental health difficulties refers to both parent-report internalizing and externalizing problems at 3 years.

**Table S3**

*Sensitivity Analysis for Sibling Bullying Roles and Trajectories of Parent-Report Mental Health Difficulties During Adolescence*

|  | **Internalizing Problems**  **Unstandardized Beta [95% CI]** | **Externalizing Problems**  **Unstandardized Beta [95% CI]** |
| --- | --- | --- |
|  |  |  |
| Sibling Bullying Involvement Group (Age 11) |  |  |
| Uninvolved | 0 [Reference] | 0 [Reference] |
| Victim-only | **1.05 [.60, 1.51]** | **1.25 [.81, 1.69]** |
| Bully-only | .76 [-.04, 1.58] | **2.01 [1.24, 2.79]** |
| Bully-victim | **1.09 [.72, 1.46]** | **1.82 [1.46, 2.18]** |
| Age | **.13 [.11, .14]** | **-.07 [-.09, -.06]** |
| Sibling Bullying Involvement Group X Age Interactions |  |  |
| Uninvolved X Age | 0 [Reference] | 0 [Reference] |
| Victim-only X Age | **-.04 [-.08, -.01]** | **-.07 [-.10, -.04]** |
| Bully-only X Age | -.04 [-.10, .02] | **-.10 [-.15, -.04]** |
| Bully-victim X Age | **-.04 [-.07, -.02]** | **-.09 [-.12, -.07]** |
| Sex |  |  |
| Girls | 0 [Reference] | 0 [Reference] |
| Boys | **-.29 [-.40, -.19]** | **.78 [.67, .89]** |
| Poverty |  |  |
| No | 0 [Reference] | 0 [Reference] |
| Yes | **.90 [.76, 1.03]** | **1.03 [.89, 1.17]** |
| Pre-existing mental health difficulties | **.34 [.32, .36]** | **.36 [.34, .37]** |

**Note.** Bold font indicates confidence intervals that did not cross zero.

**Table S4**

*The Relationships Between Sibling Bullying at age 11 Years with Self-Report Positive and Negative Mental Health at age 17 years - Coefficients for Covariates*

|  | **Model** | | | | | |
| --- | --- | --- | --- | --- | --- | --- |
| **Covariate** | **Internalizing Problems** | **Externalizing Problems** | **Psychological Distress** | **Self-Harm** | **General Wellbeing** | **Self-Esteem** |
| Sex | **-.67 [-.71, -.62]** | **.12 [.07, .17]** | **-.46 [-.51, -.42]** | **-.27 [-.32, -.21]** | **.33 [.28, .38]** | **.29 [.24, .35]** |
| Poverty | -.02 [-.07, .04] | .06 [-.01, .17] | .02 [-.05, .08] | .02 [-.04, .09] | **-.08 [-.14, -.01]** | -.04 [-.10, .03] |
| Pre-existing internalizing problems | **.05 [.02, .07]** | - | **-**.01 [-.04, .02] | -.02 [-.05, .01] | -.00 [-.03, .03] | .02 [-.01, .05] |
| Pre-existing externalizing problems | **-** | **.17 [.14, .20]** | **.07 [.04, .10]** | **.06 [.03, .10]** | **-.07 [-.10, -.04]** | **-.07 [-.10, -.05]** |

**Note.** Bold font indicates confidence intervals that did not cross zero. Values are unstandardized betas [95% confidence intervals]. Sex (0=female – reference category, 1 = male). Poverty (0= not in poverty – reference category, 1 = in poverty). For internalizing problems pre-existing mental health difficulties refers to parent-report internalizing problems at 3 years. For externalizing problems pre-existing mental health difficulties refers to parent-report externalizing problems at 3 years. For all other models, pre-existing mental health difficulties refers to both parent-report internalizing and externalizing problems at 3 years.

**Table S5**

*The Relationships Between Sibling Bullying at age 11 Years with Self-Report Positive and Negative Mental Health at age 17 years - Results of the Multivariate Regression Model*

|  | **Internalizing Problems** | **Externalizing Problems** | **Psychological Distress** | **Self-Harm** | **General Wellbeing** | **Self-Esteem** |
| --- | --- | --- | --- | --- | --- | --- |
| Sibling Bullying Involvement Group (Age 11) |  |  |  |  |  |  |
| Uninvolved | 0 [Reference] | 0 [Reference] | 0 [Reference] | 0 [Reference] | 0 [Reference] | 0 [Reference] |
| Victim-only | **.99 [.69, 1.29]** | **.59 [.37, .81]** | **1.01 [.71, 1.32]** | **.12 [.06, .18]** | **-.84 [-1.11, -.60]** | **-.48 [-.68, -.27]** |
| Bully-only | .35 [-.12, .82] | **.93 [.57, 1.29]** | **.68 [.21, 1.15]** | .08 [-.03, .20] | -.30 [-.71, .12] | -.05 [-.43, .33] |
| Bully-victim | **.65 [.41, .89]** | .**76 [.60, .92]** | **.78 [.56, 1.01]** | **.10 [05, .15]** | **-.58 [-.78, -.38]** | **-.32 [-.49, -.15]** |
| Sex |  |  |  |  |  |  |
| Female | 0 [Reference] | 0 [Reference] | 0 [Reference] | 0 [Reference] | 0 [Reference] | 0 [Reference] |
| Male | **-3.31 [-3.49, -3.12]** | **.33 [.20, .45]** | **-2.27 [-2.46, -2.07]** | **-.26 [-.30, -.23]** | **1.36 [1.18, 1.53]** | **.98 [.85, 1.11]** |
| Poverty |  |  |  |  |  |  |
| Not in poverty | 0 [Reference] | 0 [Reference] | 0 [Reference] | 0 [Reference] | 0 [Reference] | 0 [Reference] |
| In poverty | **-.23 [-.46, -.01]** | **.22 [.03, .41]** | .02 [-.21, .26] | .01 [-.04, .06] | **-.29 [-.51, -.08]** | -.12 [-.29, .05] |
| Pre-existing internalizing problems | **.05 [.01, .10]** | **-.07 [-.10, -.03]** | -.02 [-.07, .03] | -.01 [-.02, .00] | .01 [-.03, .05] | .03 [-.01, .06] |
| Pre-existing externalizing problems | **.06 [.03, .10]** | **.16 [.14, .18]** | **.09 [.06, .12]** | **.01 [.01, .02]** | **-.07 [-.10, -.04]** | **-.06 [-.08, -.04]** |

**Note.** Bold font indicates confidence intervals that did not cross zero. Values are unstandardized betas [95% confidence intervals].

**Table S6**

*The Relationship Between Transient and Repeated Sibling Bullying Victimization at age 11 and 14 years with Self-Report Positive and Negative Mental Health at age 17 years - Coefficients for Covariates*

|  | **Model** | | | | | |
| --- | --- | --- | --- | --- | --- | --- |
| **Covariate** | **Internalizing Problems** | **Externalizing Problems** | **Psychological Distress** | **Self-Harm** | **General Wellbeing** | **Self-Esteem** |
| Sex | **-.65 [-.70, -.60]** | **.14 [.09, .19]** | **-.44 [-.49, -.39]** | **-.25 [-.31, -.20]** | .**31 [.26, .35]** | **.28 [.23, .33]** |
| Poverty | -.02 [-.08, .04] | **.07 [.01, .13]** | .02 [-.05, .09] | .03 [-.04, .09] | -**.08 [-.14, -.02]** | -.03 [-.10, .03] |
| Pre-existing internalizing problems | .**05 [.02, .07]** | - | -.01 [-.03, .02] | -.02 [-.05, .01] | -.00 [-.03, .03] | .02 [-.01, .05] |
| Pre-existing externalizing problems | - | **.18 [.14, .21]** | **.07 [.04, .10]** | **.07 [.03, .10]** | **-.07 [-.10, -.04]** | -**.07 [-.11. -.04]** |

**Note.** Bold font indicates confidence intervals that did not cross zero. Values are unstandardized betas [95% confidence intervals]. Sex (0=female – reference category, 1 = male). Poverty (0= not in poverty – reference category, 1 = in poverty). For internalizing problems pre-existing mental health difficulties refers to parent-report internalizing problems at 3 years. For externalizing problems pre-existing mental health difficulties refers to parent-report externalizing problems at 3 years. For all other models, pre-existing mental health difficulties refers to both parent-report internalizing and externalizing problems at 3 years.

**Table S7**

*The Relationship Between Transient and Repeated Sibling Bullying Victimization at age 11 and 14 years with Self-Report Positive and Negative Mental Health at age 17 years- Results of the Multivariate Regression Model*

|  | **Internalizing Problems** | **Externalizing Problems** | **Psychological Distress** | **Self-Harm** | **General Wellbeing** | **Self-Esteem** |
| --- | --- | --- | --- | --- | --- | --- |
| **Victimization Group** |  |  |  |  |  |  |
| Uninvolved [0] vs Transient | **.76 [.52, .97]** | **.68 [.53, .83]** | **.72 [.51, .93]** | **.06 [.02, .11]** | **-.58 [-.76, -.40]** | **-.25 [-.40, -.10]** |
| Uninvolved [0] vs Repeated | **1.52 [1.22, 1.83]** | **1.29 [1.09, 1.48]** | **1.98 [1.68, 2.27]** | **.23 [.17, .29]** | **-1.37 [-1.62, -1.13]** | **-.80 [-1.00, -.59]** |
| Transient [0] vs Repeated | **.78 [.48, 1.07]** | **.61 [.39, .83]** | **1.26 [.96, 1.56]** | **.17 [.11, .23]** | **-.80 [-1.04, -.55]** | **-.54 [-.74, -.34]** |
| Sex |  |  |  |  |  |  |
| Female | 0 [Reference] | 0 [Reference] | 0 [Reference] | 0 [Reference] | 0 [Reference] | 0 [Reference] |
| Male | **-3.21 [-3.41, -3.05]** | **.42 [.28, .56]** | **-2.15 [-2.36, -1.94]** | **-.24 [-.28, -.21]** | **1.27 [1.10, 1.45]** | **.94 [.81, 1.07]** |
| Poverty |  |  |  |  |  |  |
| Not in poverty | 0 [Reference] | 0 [Reference] | 0 [Reference] | 0 [Reference] | 0 [Reference] | 0 [Reference] |
| In poverty | **-.21 [-.45, .04]** | **.26 [.08, .44]** | .08 [-.17, .34] | .01 [-.03, .06] | **-.31 [-.53, -.09]** | -.13 [-.31, .04] |
| Pre-existing internalizing problems | **.05 [.00, .10]** | **-.07 [-.10, -.04]** | -.02 [-.06, .02] | -.01 [-.02, .00] | .01 [-.03, .05] | .03 [-.00, .06] |
| Pre-existing externalizing problems | **.06 [.03, .09]** | **.16 [.14, .18]** | .**09 [.05, .12]** | **.01 [.01, .02]** | **-.07 [-.10, -.04]** | **-.06 [-.08, -.04]** |

**Note.** Bold font indicates confidence intervals that did not cross zero. Values are unstandardized betas [95% confidence intervals]. [0] denotes reference group

**Table S8**

*Additional Analyses to Compare Means and Trajectories of Internalizing and Externalizing Problems Amongst Sibling Bullying Groups*

|  | **Internalizing Problems**  **Unstandardized Beta [95% CI]** | **Externalizing Problems**  **Unstandardized Beta [95% CI]** |
| --- | --- | --- |
|  |  |  |
| Sibling Bullying Involvement Group |  |  |
| Victim-only [0]: Bully-only | -.22 [-1.28, .84] | .53 [-.55, 1.61] |
| Victim-only [0]: Bully-victim | .03 [-.56, .61] | **.58 [.03, 1.14]** |
| Bully-only [0]: Bully-victim | .25 [-.84, 1.33] | .05 [-.98, 1.08] |
| Sibling Bullying Involvement Group X Age Interactions |  |  |
| Victim-only [0]: Bully-only X Age | -.00 [-.08, .07] | -.01 [-.09, .07] |
| Victim-only [0]: Bully-victim X Age | -.01 [-.05, .03] | -.03 [-.07, .07] |
| Bully-only [0]: Bully-victim X Age | -.01 [-.09, .08] | -.02 [-.09, .06] |

**Note.** Bold font indicates confidence intervals that did not cross zero. [0] indicates reference category
